# Chronic Hypopituitarism Associated with Increased Postconcussive Symptoms Is Prevalent after Blast-Induced Mild Traumatic Brain Injury

**DOI:** 10.3389/fneur.2018.00072

**Published:** 2018-02-19

**Authors:** Arundhati Undurti, Elizabeth A. Colasurdo, Carl L. Sikkema, Jaclyn S. Schultz, Elaine R. Peskind, Kathleen F. Pagulayan, Charles W. Wilkinson

**Affiliations:** ^1^Department of Psychiatry and Behavioral Sciences, University of Washington, Seattle, WA, United States; ^2^Geriatric Research, Education and Clinical Center (GRECC), VA Puget Sound Health Care System, U.S. Department of Veterans Affairs, Seattle, WA, United States; ^3^VISN 20 Northwest Network Mental Illness Research, Education, and Clinical Center (MIRECC), VA Puget Sound Health Care System, U.S. Department of Veterans Affairs, Seattle, WA, United States

**Keywords:** traumatic brain injury, blast, concussion, pituitary, military, posttraumatic stress disorder, growth hormone deficiency, veterans

## Abstract

The most frequent injury sustained by US service members deployed to Iraq or Afghanistan is mild traumatic brain injuries (mTBI), or concussion, by far most often caused by blast waves from improvised explosive devices or other explosive ordnance. TBI from all causes gives rise to chronic neuroendocrine disorders with an estimated prevalence of 25–50%. The current study expands upon our earlier finding that chronic pituitary gland dysfunction occurs with a similarly high frequency after blast-related concussions. We measured circulating hormone levels and accessed demographic and testing data from two groups of male veterans with hazardous duty experience in Iraq or Afghanistan. Veterans in the mTBI group had experienced one or more blast-related concussion. Members of the deployment control (DC) group encountered similar deployment conditions but had no history of blast-related mTBI. 12 of 39 (31%) of the mTBI participants and 3 of 20 (15%) veterans in the DC group screened positive for one or more neuroendocrine disorders. Positive screens for growth hormone deficiency occurred most often. Analysis of responses on self-report questionnaires revealed main effects of both mTBI and hypopituitarism on postconcussive and posttraumatic stress disorder (PTSD) symptoms. Symptoms associated with pituitary dysfunction overlap considerably with those of PTSD. They include cognitive deficiencies, mood and anxiety disorders, sleep problems, diminished quality of life, deleterious changes in metabolism and body composition, and increased cardiovascular mortality. When such symptoms are due to hypopituitarism, they may be alleviated by hormone replacement. These findings suggest consideration of routine post-deployment neuroendocrine screening of service members and veterans who have experienced blast-related mTBI and are reporting postconcussive symptoms.

## Introduction

“Concussions,” a term often used synonymously with “mild traumatic brain injuries” (mTBI), accounted for 2.8 million emergency room visits or hospitalizations in the US in 2013 (1). The number of reported concussions has increased especially rapidly in high school, college, and professional athletes and deployed service members. Approximately 75% of diagnosed TBIs are mTBIs (2). In response, the focus of research and media attention on the prevalence, natural history, treatment, and prevention of mTBI in athletes has increased dramatically during the past decade. However, mTBI in military personnel and veterans has received less public notice, even though mTBI—most often caused by blasts from improvised explosive devices—is the most frequent injury sustained by US troops deployed to Iraq and Afghanistan (3–5). mTBIs constituted 82.4% of approximately 290,000 military TBIs diagnosed from 2000 to 2013. In both civilian and military populations, many mTBIs are unreported or undiagnosed, and the true incidence has been estimated to be two to five times higher than current reports (6–9).

The American Congress of Rehabilitation Medicine has defined mTBI as manifested by at least one of the following: 1. a period of loss of consciousness (LOC) of approximately 30 min or less; 2. loss of memory not greater than 24 h for events immediately before or after the incident; 3. any alteration in mental state at the time of the incident (e.g., feeling dazed, disoriented, or confused); or 4. focal neurological deficit(s) that may or may not be transient (10, 11).

A frequent consequence of TBI is chronic hypopituitarism, defined as a deficiency in one or more pituitary hormone axes. Since 2000, more than 40 research papers have characterized chronic neuroendocrine deficiencies resulting from TBI, more than half of which reported a prevalence of 25–50% after TBIs from all causes. Although review articles often assert an association of the prevalence of hypopituitarism with the severity of the injury, multiple studies that included a range of TBIs from mild to severe have found no relationship between pituitary dysfunction and severity of TBI (12–21). Repetitive mild TBIs have been shown to result in a high prevalence of hypopituitarism in several studies (22–27).

We previously published preliminary data supporting the high prevalence of chronic hypopituitarism in US military veterans deployed to Iraq or Afghanistan who sustained one or more blast-related concussions compared to similarly deployed veterans without blast exposure (27). We have now extended our preliminary findings to a larger sample and have also examined the effects of chronic hypopituitarism on measures of mood, sleep quality, symptoms of posttraumatic stress disorder (PTSD), and cognitive functioning.

## Materials and Methods

### Participants and Sample Acquisition

The Congressionally Directed Medical Research Programs Concept Award that funded this study prohibited direct sampling of biological fluids from human participants and access to identifiable private information. Therefore, all plasma and serum samples, demographic, and blast exposure data used in the study were obtained from an established biorepository entitled “Alzheimer’s Disease Research Center Participant Registry and Sample Repository.” The VA Puget Sound Health Care System Institutional Review Board and the U.S. Army Medical Research and Materiel Command Office of Research Protections Human Research Protection Office approved the subject protocol with a waiver of informed consent. All participants whose samples were utilized had provided written informed consent to have their samples and data used in future research of this type. No direct intervention with any participant was allowed. These conditions precluded the use of provocative testing as a part of the screening procedure.

Plasma and serum specimens and demographic and testing data were obtained from the repository for 59 male veterans with documented hazardous duty experience in Iraq and/or Afghanistan with the US Armed Forces. Thirty-nine of these individuals (the mTBI group) had sustained at least one explosive blast-induced concussion. The remaining 20 veterans (deployment control or DC group) were exposed to similar deployment conditions but had not experienced a blast-related mTBI.

Blood samples from 95 healthy male community volunteers with no evidence or history of cognitive or functional decline and no history of military service or TBI were also retrieved from the repository. These samples were used only for the establishment of normative hormone concentration ranges using our assay methods.

### Exclusion Criteria and Screening

Exclusion criteria for all participants included a history of TBI with LOC greater than 30 min; penetrating head wound; seizure disorder; insulin-dependent diabetes; current or past diagnosis of schizophrenia, other psychotic disorders, bipolar disorder, or dementia with Diagnostic and Statistical Manual of Mental Disorders, Fourth Edition (DSM-IV) criteria (28); or a DSM-IV diagnosis of alcohol or other substance abuse or dependence within the previous 3 months.

Screening for study eligibility included physical and neurological examinations. The Structured Clinical Interview for DSM-IV (SCID-IV) was used to screen for exclusionary mood, psychotic, anxiety, and substance abuse disorders (29).

### Demographics and Military History

Demographic, military deployment history, and blast exposure information were collected from all participants at screening. Data collected included age, education (in years), race, body mass index (BMI), number of deployments to Iraq or Afghanistan, total deployment time (in months), number of blast exposures during deployment, and time since last blast exposure.

Blast exposure and mTBI histories were obtained from participants in a semi-structured clinical interview by two expert clinicians. Specific inquiries were made about total number of blast-related mTBIs in Iraq or Afghanistan and lifetime history of non-blast head injuries accompanied by acute symptoms of mTBI. Evaluations based on these interviews determined the assignment of the participants to mTBI or DC groups.

The Wechsler Test of Adult Reading (WTAR) and the Combat Experiences Questionnaire (CEQ) were also administered. The WTAR is a measure of premorbid intellectual functioning that is thought to be resilient to mTBI. The test provides estimates of both verbal IQ and the overall level of general cognitive and intellectual functioning (Full Scale IQ) (30, 31). The CEQ is an 18-item true/false questionnaire excerpted from Hoge et al. (32) addressing the frequency and severity of experiences that participants may have been exposed to during deployment (e.g., shooting or directing fire at the enemy; being attacked, or ambushed).

### Hormone Measurement

Blood samples were collected between 9:00 a.m. and 10:00 a.m. from supine participants 30 or more min after the insertion of an intravenous catheter in an antecubital vein. Samples for determination of hormones in plasma were collected in chilled tubes containing ethylenediaminetetraacetic acid, placed on ice, and centrifuged at 4°C prior to removal of the plasma fraction. Blood samples for serum hormone quantification were collected in serum-separator tubes, allowed to clot at room temperature for 10 min, and centrifuged at 4°C to isolate serum. Serum and plasma samples were aliquoted and stored at −70°C. Eleven pituitary or target-organ hormones were measured in these samples. The type, source, and performance characteristics of the assay kits used for the measurements are shown in Table 1. Adrenocorticotropin (ACTH), cortisol, thyroid-stimulating hormone (TSH), oxytocin, and vasopressin concentrations were determined in plasma; free thyroxine, luteinizing hormone (LH), follicle-stimulating hormone (FSH), prolactin, total testosterone, and insulin-like growth factor-I (IGF-I) were measured in serum. Measurements of plasma and urine osmolality, plasma and urine Na+, plasma K+, urine specific gravity (USG), blood urea nitrogen, creatinine, and glucose were used in determining functional vasopressin insufficiency.

**Table 1:** Assay kit sources and characteristics.

Hormone	Kit name			Manufacturer	Location	
Adrenocorticotropin (ACTH)	ACTH IRMA			Scantibodies Laboratory	Santee, CA, USA	
Cortisol	Corti-Cote Cortisol RIA			MP Biomedicals	Santa Ana, CA, USA	
Follicle-stimulating hormone (FSH)	DELPHIA hFSH Fluoroimmunoassay			PerkinElmer	Waltham, MA, USA	
Insulin-like growth factor (IGF-I)	Human IGF-I Quantikine ELISA			R&D Systems	Minneapolis, MN, USA	
Luteinizing hormone (LH)	ImmuChem Coated Tube LH ^125^I RIA			MP Biomedicals	Santa Ana, CA, USA	
Oxytocin	Oxytocin ELISA			Enzo Life Sciences, Inc.	Farmingdale, NY, USA	
Prolactin	ImmuChem Coated Tube Prolactin ^125^I IRMA			MP Biomedicals	Santa Ana, CA, USA	
Testosterone	ImmuChem Double Antibody Testosterone ^125^I RIA			MP Biomedicals	Santa Ana, CA, USA	
Thyroxine	Free Thyroxine (FT_4_) Immunoassay			MP Biomedicals	Santa Ana, CA, USA	
Thyroid-stimulating hormone (TSH)	ImmuChem Coated Tube TSH ^125^I IRMA			MP Biomedicals	Santa Ana, CA, USA	
Vasopressin	Vasopressin Direct RIA			Buhlmann Diagnostics	Amherst, NH, USA	

**Hormone**	**Assay type**	**Sample type**	**Assay size**	**Sample size (µl)**	**Assay range**	**Sensitivity**

ACTH	IRMA	Plasma	100 tubes	200	2–372 pmol/L	0.22 pmol/L
Cortisol	RIA	Plasma	100 tubes	25	0.027–1.65 µmol/L	5.79 nmol/L
FSH	Fluoroimmunoassay	Serum	96 wells	25	0.98–256 IU/L	0.05 IU/L
IGF-I	ELISA	Serum	96 wells	0.5	0.02–1.31 nmol/L	2.62 pmol/L
LH	RIA	Serum	100 tubes	100	2.5–200 mIU/mL	1.5 mIU/mL
Oxytocin	ELISA	Plasma	96 wells	400	0–504 pmol/L	5.2 pmol/L
Prolactin	IRMA	Serum	100 tubes	25	0.11–4.35 nmol/L	109 pmol/L
Testosterone	RIA	Serum	100 tubes	50	0.69–55.5 nmol/L	0.14 nmol/L
Thyroxine	EIA	Serum	96 wells	50	5.8–98 pmol/L	6.44 pmol/L
TSH	IRMA	Plasma	100 tubes	200	0.2–50 IU/L	0.04 IU/L
Vasopressin	RIA	Plasma	100 tubes	400	1.15–73.84 pmol/L	0.09 pmol/L

*IRMA, immunoradiometric assay; RIA, radioimmunoassay; ELISA, enzyme-linked immunosorbent assay; EIA, enzyme immunoassay*.

### Criteria for Identifying Pituitary Hormone Deficiencies

Concentration percentiles based on the lognormal distribution of each hormone in blood samples from 95 community control participants were calculated. Dysfunction in each of five anterior pituitary axes and two posterior pituitary axes were defined by specific percentile levels based on the consensus of criteria used in multiple published studies of pituitary dysfunction after TBI from all causes. Due to concerns about the reliability and sensitivity of the vasopressin assay, measures of urine and plasma osmolality (Posm) and electrolytes were the primary determinants of diabetes insipidus (DI) and syndrome of inappropriate antidiuretic hormone secretion. Measurement of serum IGF-I concentration was used for growth hormone deficiency (GHD) screening. Since IGF-I concentrations decline significantly with increasing age in adults, the lognormal distribution of IGF-I levels in the reference group was adjusted for age, and the IGF-I concentration of each of the TBI and DC subjects was plotted in relation to age to provide appropriate comparisons. The criteria for deficient (or excessive) hormone levels in each axis are shown in Table 2.

**Table 2:** Screening criteria for identifying abnormal circulating hormone levels.

Cutoff criteria based on lognormal distribution of community control reference sample			
Disorder	Hormone	Percentile	Cutoff (SIU)
Adrenal insufficiency	Cortisol	<10th percentile	141.8 nmol/L
	Adrenocorticotropin	<10th percentile	3.4 pmol/L
Thyroid deficiency	Free thyroxine	<5th percentile	11.97 pmol/L
	Thyroid-stimulating hormone	<50th percentile	1.78 mlU/L
Hypogonadism	Total testosterone and either luteinizing hormone (LH) or follicle-stimulating hormone (FSH)	<5th percentile <10th percentile	6.9 nmol/L LH: 1.32 IU/L, FSH: 1.63 IU/L
	OR total testosterone and prolactin	<5th percentile >95th percentile	6.9 nmol/L 910.86 pmol/L
Hypo-/Hyperprolactinemia	Prolactin	<5th percentile	189.56 pmol/L
		OR > 95th percentile	910.86 pmol/L
Growth hormone deficiency	Insulin-like growth factor	<age-adjusted 10th PCTL	(SDS < −1.4)
Hypooxytocinemia	Oxytocin	<5th percentile	1.8 pmol/L
Diabetes insipidus	Vasopressin and Posm ≫ Uosm	<5th percentile USG < 1.005	0.25 pmol/L

Hypopituitarism—abnormalities in at least one of these seven axes			

*Posm, plasma osmolality; Uosm, urine osmolality; PCTL, percentile; USG, urine specific gravity*.

### PTSD Evaluation

The Clinician-Administered PTSD Scale for DSM-IV (CAPS) (33) is a structured interview designed to make a categorical diagnosis of PTSD and to assess the frequency and intensity of 17 PTSD symptoms to yield continuous severity scores for each symptom and the disorder as a whole. Administration of the CAPS requires identification of an index traumatic event to serve as the basis for symptom inquiry. In the absence of an appropriate traumatic event the symptom severity questionnaire is not administered.

### Symptom Self-Report Questionnaires

The PTSD Checklist-Military Version (PCL-M), a self-report inventory of the 17 DSM-IV symptoms that define PTSD, was used to assess individual PTSD symptoms (34). The Neurobehavioral Symptom Inventory (NSI) is a 22-item questionnaire designed to assess the presence and severity of common cognitive, emotional, sensory, and somatic postconcussive symptoms (35). The Patient Health Questionnaire-9 (PHQ-9) is the 9-item depression module of the Patient Health Questionnaire that corresponds with DSM-IV criteria for depression (36). The Pittsburgh Sleep Quality Index (PSQI) is a self-report questionnaire assessing sleep quality and disturbances over a 1-month time interval (37). The Alcohol Use Disorders Identification Test-Consumption enquires about frequency and quantity of typical alcohol consumption and the frequency of episodes of heavy drinking (38).

### Cognitive Measures

#### Trail Making Test

The Trail Making Test (TMT) consists of two parts. Trails A is a measure of processing speed (visual search and motor speed skills). Trails B has an added set-shifting component and is widely regarded as a measure of executive functioning (39). The score on each part represents the amount of time required to complete the task. Normative scores, corrected for age, education, race, and gender, are reported as *T*-scores.

#### Ruff 2&7 Selective Attention Test

The Ruff 2&7 Test is a measure of visual sustained and selective attention. Participants identify and mark target digits that are intermixed with other number distractors or capital letter distractors. This measure yields age and education corrected Total Speed (total target numbers crossed out) and Total Accuracy *T* scores. This measure has been shown to be sensitive to the effects of mTBI (40).

#### Test of Memory Malingering (TOMM)

The TOMM was used to evaluate performance validity on the neuropsychological measures in this study (41). This measure involves two learning/recognition trials and an optional retention trial following a delay. Established cutoff scores were used to determine performance validity; if participants scored below these cutoffs, their neuropsychological test scores were not included in the analyses.

### Statistical Analysis

Analysis of the difference between the proportion of participants with hypopituitarism in the DC and mTBI groups was performed with a modified “N-1” chi-squared test for 2 × 2 contingency tables (42, 43). Analysis of the difference in the number of individual hormonal abnormalities between the two groups was performed with the unequal variance *t*-test for independent samples (44). Analysis of the relationships of mTBI and hypopituitarism with demographic, military history, symptom self-report, and cognitive testing data were performed with two-way analysis of variance (ANOVA). One-way ANOVAs were used to analyze the differences in the scores among the four participant subgroups, DC-N, DC-HP, mTBI-N, and mTBI-HP, on each of the individual items of the NSI.

## Results

### Identification of Abnormal Plasma/Serum Hormone Concentrations

**Figure 1:**
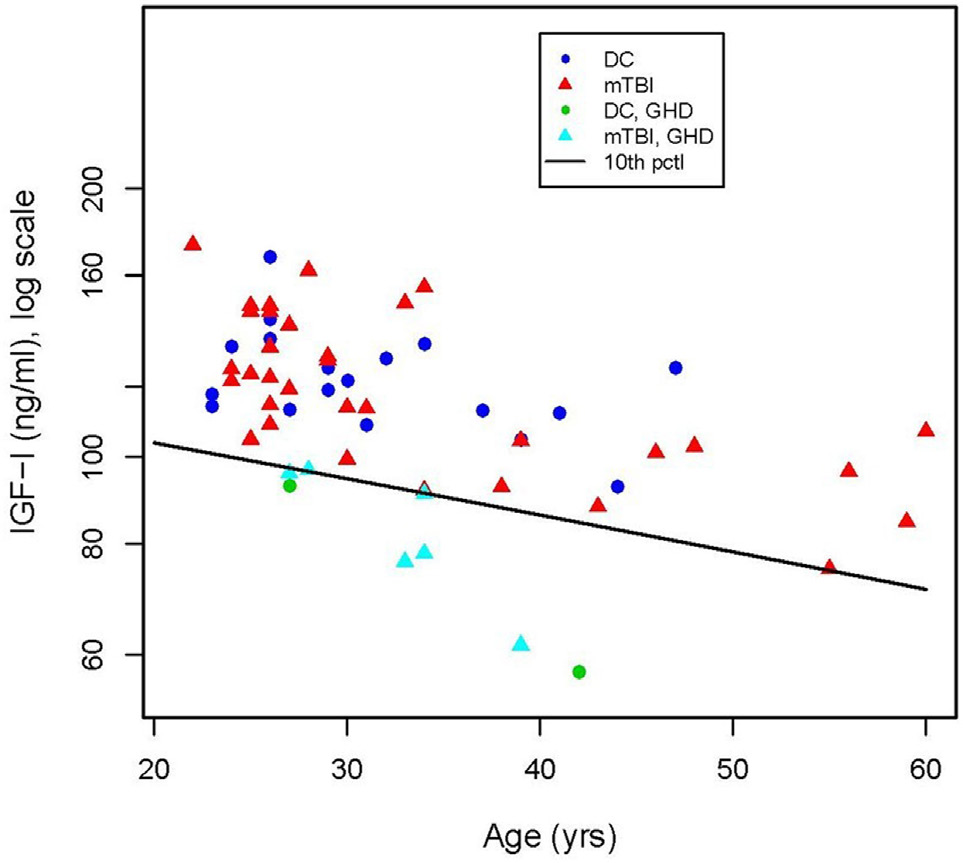
Concentration of insulin-like growth factor (IGF-I) in serum of deployment control (DC) (circles) and mild traumatic brain injuries (mTBI) (triangles) participants as a function of age. The criterion for a positive screen for growth hormone is an IGF-I level below the age-adjusted 10th percentile of IGF-I concentration (diagonal line) in the community control reference group. Serum IGF-I values of six of the mTBI group (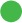) and two of the DC group (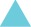) fell below the cutoff line.

12 of the 39 mTBI participants (31%) and 3 of the 20 DC participants (15%) screened positive for one or more hormonal disorders. Of the 15 participants with abnormal hormone levels, 8, including 2 of the DC participants, had markedly low basal serum IGF-I levels consistent with GHD. The diagonal line in Figure 1 represents the age-adjusted 10th percentile of the IGF-I concentration of the community control reference sample [equivalent to an SD score (SDS) of −1.4] used as the cutoff level for identifying probable GHD (Table 2). Basal IGF-I concentrations of four of the eight (mTBI-E, mTBI-A, mTBI-B, and DC-A) participants who screened positive fell below an SDS of −2.0 relative to the age-adjusted mean.

Hypogonadism was identified in two participants in the mTBI group. Two mTBI participants screened positive for hypothyroidism and one participant in each group screened positive for hyperprolactinemia. No participants screened positive for secondary adrenal insufficiency (sAI) (Table 3).

**Table 3:** Hormonal disorders and self-report questionnaire percentiles for individual participants who screened positive for hypopituitarism.

Identifiers	Deficiency	PCL-M	NSI	PHQ-9	PSQI	AUDIT-C
Mild traumatic brain injuries (mTBI)-A	GHD	98	92	98	90	22
mTBI-B	GHD	82	88	83	97	85
mTBI-C	Diabetes insipidus (DI)	55	56	38	97	5
mTBI-D	Hypothyroidism, DI	94	97	98	57	85
mTBI-E	GHD, hypogonadism, hyperprolactinemia	78	69	72	NS	38
mTBI-F	Hypothyroidism	98	99	98	35	11
mTBI-G	Hypogonadism	98	99	97	87	99
mTBI-H	Hypooxytocinemia	74	60	53	29	56
mTBI-I	GHD	49	39	29	23	11
mTBI-J	GHD	18	45	25	23	72
mTBI-K	DI	51	35	21	87	93
mTBI-L	GHD, hypooxytocinemia	22	22	14	23	97
DC-A	GHD	57	37	58	57	56
DC-B	GHD	10	12	14	5	56
DC-C	Hyperprolactinemia	53	41	58	64	11

*Percentile is based on the ranking of all participants. The higher the percentile, the more severe or frequent the symptoms endorsed. The participant identifiers in the far-left column are “dummy” identifiers and are not the participant identification sequences used in the study. Percentile values falling in the highest (fourth) quartile of scores are highlighted in yellow*.

*GHD, growth hormone deficiency; PCL-M, Posttraumatic Stress Disorder CheckList-Military; NSI, Neurobehavioral Symptom Inventory; PHQ-9, Patient Health Questionnaire-9; PSQI, Pittsburgh Sleep Quality Index; AUDIT-C, Alcohol Use Disorders Identification Test-Consumption; NS, no score*.

Five mTBI group individuals screened positive for posterior pituitary hormone deficiencies. Three were identified with DI based on multiple measures (including plasma and urine Na+, plasma and urine osmolality, and USG), and two screened positive for hypooxytocinemia. One individual, mTBI-E, screened positive for GHD, hypogonadism, and hyperprolactinemia. mTBI-D screened positive for hypothyroidism and DI, and TBI-L had hormone concentrations indicative of both GHD and hypooxytocinemia.

The proportion of individuals in each participant group who screened positive for one or more indices of hypopituitarism did not differ significantly (*p* = 0.094) when compared using the modified “N-1” chi-squared test for 2 × 2 contingency tables (42, 43).

When the differences between the two groups in the number of individual deficiencies were compared, a significant group difference was observed. The 39 members of the mTBI group screened positive for a total of 16 individual pituitary disorders (mean number of deficits per person = 0.413, SD = 0.715). Three of 20 (15%) DC participants showed evidence of a single pituitary impairment (mean = 0.15, SD = 0.366). There was a significant difference in total number of deficiencies between the groups by *t*-test of independent samples with unequal variances (*t* = 1.85, df = 56.99, *p* = 0.035) (44).

### Relationships of mTBI and Hypopituitarism to Demographics and Military History

After hormonal screening, the mTBI and DC groups were divided into subgroups based on the presence or absence of hypopituitarism. The resulting four groups are: deployment controls with (DC-N) and without (DC-HP) normal pituitary function, and mTBI group members with (mTBI-N) and without (mTBI-HP) normal pituitary function. Differences in demographics, deployment history, and blast exposure of the subgroups were analyzed with two-way ANOVA (Table 4). At the time of study enrollment, the four groups of veteran participants did not differ significantly in age, education, BMI, number of deployments, total months deployed, or time since last the blast-related concussion. There also were no significant differences among subgroup scores on the WTAR. There were, however, significant group differences in combat experiences endorsed on the CEQ. Both mTBI (*F* = 90.9, df = 1, *p* < 0.0001) and hypopituitarism (*F* = 4.21, df = 1, *p* < 0.05) were significantly associated with increased numbers of combat experiences.

**Table 4:** Subgroup means ± SEM and ranges ( ) for demographic characteristics, deployment history, and blast exposure of study participants.

Demographics	DC-N	DC-HP	TBI-N	TBI-HP
Age	31.8 ± 1.79 (23–47)	32.7 ± 4.7 (27–42)	32.1 ± 2.19 (22–60)	35.8 ± 2.08 (27–48)
Education (years)	13.8 ± 0.38 (12–17)	13.3 ± 0.67 (12–14)	14.3 ± 0.36 (12–20)	14.2 ± 0.3 (12–16)
Race	13/17 White, 1/17 Black, 3/17 other	3/3 White	20/27 White, 2/27 Black, 2/27 Asian, 1/27 Native Hawaiian, 2/27 other	8/12 White, 1/12 Black, 3/12 other
BMI	27.1 ± 1 (21–34.7)	25.4 ± 3.05 (19.8–30.3)	27.8 ± 0.84 (20.5–35.5)	30.9 ± 1.5 (20.1–38.7)
WTAR—Verbal IQ	108.06 ± 2.17	114.5 ± 2.5	106.59 ± 1.91	106.17 ± 2.26
WTAR—Full Scale IQ	107.41 ± 2.05	113.5 ± 2.5	105.89 ± 1.93	105.58 ± 2.17
Combat Experiences Questionnaire	4.7 ± 1.0 (0–14)	4.3 ± 2.3 (0–8)	12.9 ± 0.5 (6–18)	13.6 ± 0.7 (9–17)

**Deployment History**	**DC-N**	**DC-HP**	**TBI-N**	**TBI-HP**

Number of deployments	2 (1–3)	2 (2–3)	2 (1–3)	2 (1–3)
Total deployment time (months)	14.1 months ± 1.23 (7.1–24.8)	28.7 months ± 5.03 (19.3–36.5)	19.2 months ± 2.03 (5.1–48.7)	21.9 months ± 2.3 (12–39.5)

**Blast exposure**	**DC-N**	**DC-HP**	**TBI-N**	**TBI-HP**

Number of deployment blast exposures	0	0	8.48 ± 2.12 (1–52)	14.58 ± 5.18 (3–66)
Time since last blast exposure	0	0	4.61 years ± 0.39 (1.65–8.19)	4.29 years ± 0.49 (1.65–8.19)

*BMI, body mass index; WTAR, Wechsler Test of Adult Reading*.

### PTSD Diagnosis

Two of 17 participants (11.8%) in the DC-N group, 1 of 3 (33.3%) in the DC-HP group, 13 of 26 (50.0%, one unscored) in the mTBI-N group, and nine of 12 (75.0%) in the mTBI-HP group were clinically diagnosed with PTSD with the CAPS. There was no significant difference on the total CAPS score between the mTBI-N and mTBI-HP groups (Table 5) (33). The scores of the two members of the DC-N group and one of the DC-HP group who were diagnosed with PTSD were not entered in the table because the Ns were too small to include in the ANOVA.

**Table 5:** Means and SEMs of participant group scores on symptom self-report questionnaires and cognitive instruments.

Participant group	DC-N (*n* = 17)	DC-HP (*n* = 3)	mTBI-N (*n* = 27)	mTBI-HP (*n* = 12)	mTBI main effect	HP Main
**Self-report Questionnaires**						
CAPS posttraumatic stress disorder (PTSD) Diagnosis	2/17 11.8%	1/3 33.3%	14/27 51.9%	9/12 75.0%	*p* < 0.005	*p* < 0.05
CAPS Total Score	–	–	53.3 ± 6.1	65.0 ± 9.4	–	*p* = 0.291
PTSD CheckList-Military	26.1 ± 3.2	35.0 ± 9.0	46.2 ± 3.1	54.0 ± 5.7	*p* < 0.0001	*p* < 0.02
Neurobehavioral Symptom Inventory	8.4 ± 2.6	11.3 ± 5.7	26.9 ± 3.2	37.1 ± 6.6	*p* < 0.0001	*p* < 0.02
Patient Health Questionnaire-9	3.9 ± 1.3	6.7 ± 3.3	9.7 ± 1.4	12.7 ± 2.9	*p* < 0.005	*p* = 0.076
Pittsburgh Sleep Quality Index	5.8 ± 1.0	7.0 ± 3.5	10.4 ± 1.1	11.1 ± 1.7	*p* < 0.005	*p* = 0.308
AUDIT-C	2.7 ± 0.4	3.0 ± 1.0	4.1 ± 0.4	4.2 ± 0.9	*p* < 0.05	*p* = 0.369
**Cognitive measures**						
Trail making test trails A	55.0 ± 2.5	69.0 ± 4.0	55.6 ± 2.0	54.4 ± 3.4	*p* = 0.665	*p* = 0.700
Trail making test trails B	52.7 ± 3.2	63.0 ± 4.0	48.1 ± 2.8	47.8 ± 3.3	*p* = 0.128	*p* = 1.000
Ruff 2 & 7—total speed	50.1 ± 2.3	59.5 ± 1.5	50.6 ± 1.7	46.5 ± 2.3	*p* = 0.462	*p* = 0.496
Ruff 2 & 7—total accuracy	47.1 ± 2.2	51.0 ± 4.0	45.8 ± 2.1	46.3 ± 2.9	*p* = 0.742	*p* = 0.731

*Higher scores on symptom self-report questionnaires reflect endorsement of more severe and/or frequent symptoms. Higher scores on the cognitive tests indicate better performance. The fractions in the “CAPS PTSD Diagnosis” row of the table indicate the number of participants with a clinical diagnosis of PTSD in each group divided by the total number of participants in the group. The two columns to the far right indicate the level of significance of the main effects of mild traumatic brain injury (mTBI Main) and hypopituitarism (HP Main) on group scores on each instrument by two-way analysis of variance. One mTBI-N and one mTBI-HP participant scored below the cutoff level on the Test of Memory Malingering, and their scores have been deleted from the statistical computations for cognitive measures. Probability values < 0.02 are printed in red to indicate statistical significance of main effects*.

*CAPS, Clinician-Administered PTSD Scale for DSM-IV; AUDIT-C, alcohol use disorders identification test-consumption*.

There were significant main effects of mTBI on each of five self-report questionnaires by two-way ANOVA (Table 5) and significant main effects of hypopituitarism on two of the questionnaires: those measuring PTSD symptoms and postconcussive symptoms. The mean score of the mTBI-HP group was numerically highest of the four groups on the total score of each of the self-report questionnaires.

Scores and percentile rankings on each of the self-report questionnaires were examined for individuals who screened positive for hypopituitarism to assess whether specific neuroendocrine disorders might be uniquely associated with extreme scores on a particular questionnaire (Table 3). Due to the small number of individuals in each category, it is not possible to come to any definitive conclusions. However, some interesting patterns appeared when percentiles were examined. For example, each of the two veterans who screened positive for hypothyroidism scored in the fourth quartile of the self-report questionnaires assessing PTSD symptoms, depression symptoms, postconcussive symptoms, and current alcohol use symptoms. One of the two participants reported sleep inefficiency in the fourth quartile. Of a total of eight participants in the mTBI-HP and DC-HP groups that screened positive for GHD, only two in the mTBI group, mTBI-A and mTBI-B, had scores in the fourth quartile on more than one symptom self-report questionnaire. The IGF-I serum concentrations of both fell more than 2 SDS below the age-adjusted mean. No members of the DC-HP group scored in the fourth quartile of any self-report questionnaires.

**Figure 2:**
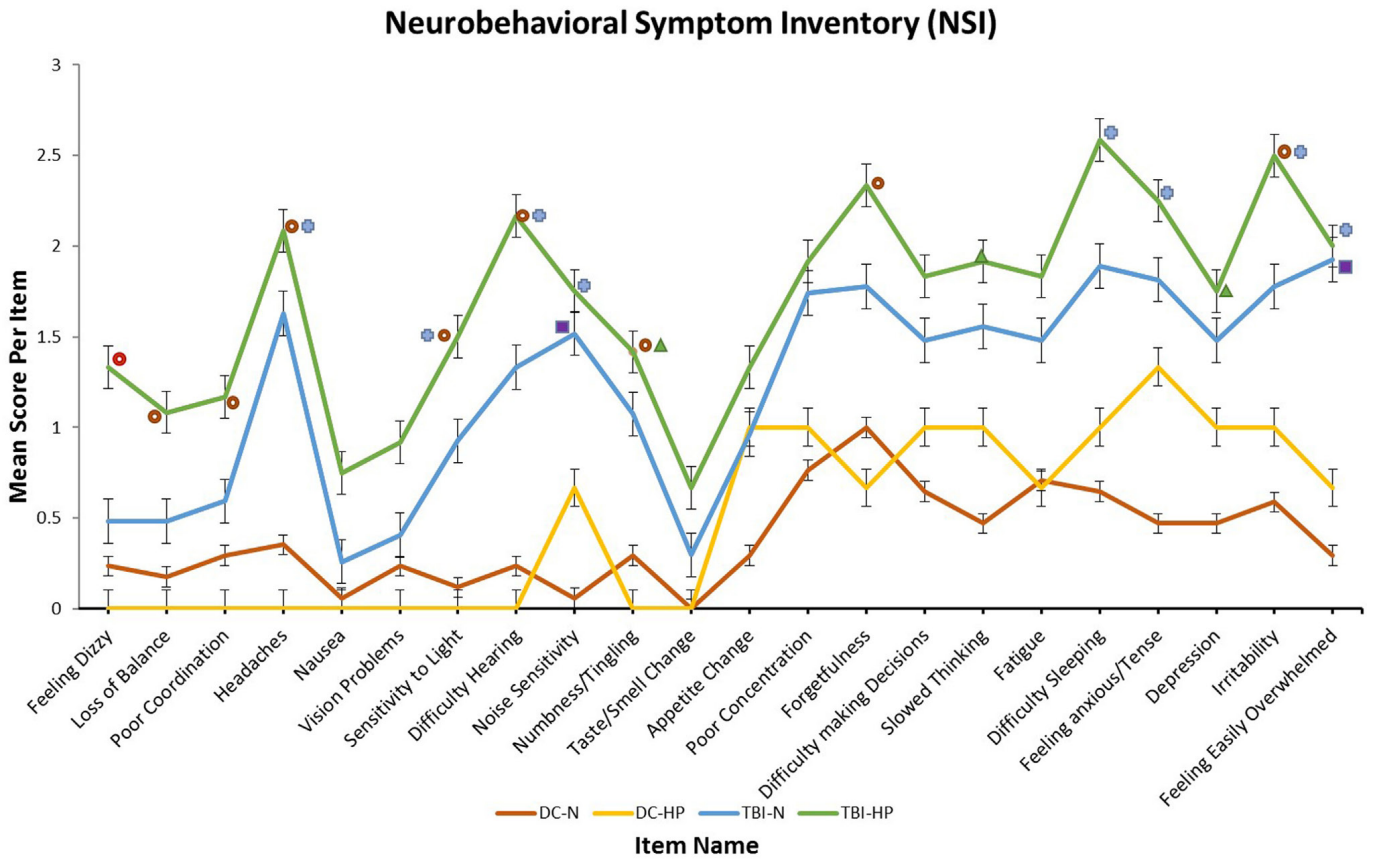
Mean scores of each participant group on each item of the neurobehavioral symptom inventory. T-bars indicate SEMs. The higher the score, the greater the frequency or severity of the symptom endorsed. All symbols indicate a significant main effect of participant group on that self-report item by one-way analysis of variance (ANOVA). Significant group effects were found by one-way ANOVA on 15 of 22 items. Each symbol represents a specific result of *post hoc* Tukey HSD tests. 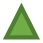 No significant paired comparisons by Tukey HSD. 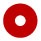 Significant difference between mTBI-HP and DC-N by Tukey HSD. 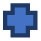 Significant difference between mTBI-HP and DC-HP by Tukey HSD. 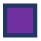 Significant difference between mTBI-N and DC-HP by Tukey HSD.

Significant group effects were found on 15 of 22 of the individual items on the NSI by one-way ANOVA. The mean score of the mTBI-HP group was the highest on all 22 of the items, as illustrated in Figure 2. The highest scores of the mTBI-HP group on the individual components of the NSI were on the “forgetfulness,” “difficulty sleeping,” and “irritability” items.

### Cognitive Measures

No significant participant group differences were found by two-way ANOVA of the total scores of any of the cognitive measures used in the study. Neither mTBI nor hypopituitarism had a measurable influence on the limited number of cognitive domains explored with these instruments, i.e., processing speed (TMT: trails A–*T* score), mental flexibility (TMT: trails B–*T* score), or selective attention (Ruff 2&7: total speed *T* score, total accuracy *T* score) (Table 5). All participants but two scored above the published cutoff for performance validity on the TOMM, suggesting that their performance on neuropsychological measures was an accurate reflection of their current cognitive abilities. One mTBI-N and one mTBI-HP participant scored below the cutoff level, and their scores have been deleted from the statistics for cognitive measures shown in Table 5.

## Discussion

Our findings support the hypothesis that the prevalence of chronic posttraumatic hypopituitarism (PTHP) consequent to blast-related mTBI in US Armed Forces veterans is in accord with that reported in studies of pituitary dysfunction in the general population after TBI from all causes. Twelve of 39 (31%) deployed veterans who sustained blast-related concussions and three veterans in the DC group screened positive for hypopituitarism. A wide range of values (from 5 to 90%) for the prevalence of PTHP in civilians has been reported in 40-plus studies since 2000 with most finding a range of 25–50% (27, 45). The large variance is due to differences in the populations sampled, injury severity, time since injury, hormone measurement methods, and screening and clinical diagnostic criteria (46, 47). We employed clearly defined screening criteria calculated from the distribution of basal morning concentrations of each hormone in a reference population.

The hormone concentration measurements obtained in this study are valuable as screening assessments but cannot be viewed as diagnostic in the absence of provocative testing and/or clinical evaluation. They can, however, serve to direct the focus of clinical appraisal by identifying individuals most likely to suffer from clinically significant deficits. Hypogonadism, hypothyroidism, and lactotroph dysfunction can be provisionally identified by measuring basal hormone levels. However, central DI resulting from vasopressin deficiency depends upon determination of USG, electrolyte concentrations, urine and Posm, and performance of a water deprivation test for adequate screening. Although measurement of basal IGF-I, ACTH, and cortisol levels are helpful screening tools, diagnosis of GHD and sAI require provocative testing for accurate diagnosis. Since this study was restricted to the use of banked samples, provocative testing was not possible.

The finding that three of the participants in the DC group screened positive for an endocrine disorder—two for GHD and one for hyperprolactinemia—was not unexpected. First, there is a possibility that these individuals had sustained an unrecognized mTBI in their past. The DC participants whose blood samples and data were drawn from the repository for the study had undergone thorough screening and detailed interviews about their TBI history at the time of sample collection and denied having experienced a concussion. However, given the relative lack of knowledge about concussions and the unconcern about “getting your bell rung” prevalent 20 or more years ago, when these veterans were growing up, the existence of prior mTBIs from impact cannot be ruled out absolutely in either group. However, the study’s focus was on blast-related mTBI, the mechanisms of which differ substantially from those of impact mTBI (48, 49), and the screening process eliminated the possibility of prior blast-related concussions in the DC group.

Second, as stated above, hormonal screening does not provide conclusive evidence of clinically significant deficiencies in the absence of provocative testing and/or evaluation of potential symptoms. Also, the use of specific concentration cutoff criteria to identify deficiencies does not take into account relevant variables such as time of day, age, gender, body weight, or fasting state. Participant DC-C screened positive for hyperprolactinemia by exceeding our normal prolactin concentration range by only a fraction of a percentile, clearly an insufficient basis for a clinical diagnosis of hyperprolactinemia.

As is characteristic of previous studies of PTHP, we found that positive screens for GHD occurred more frequently than those for any other pituitary hormone deficiency. Single daytime measurements of serum GH are not valid indicators of somatotroph function or daily GH release. Approximately 75% of GH secretion occurs during nighttime sleep, and concentrations are markedly low during the day, with short, irregularly spaced pulses of secretion occurring at long intervals (50). However, GH-stimulated hepatic production of IGF-I can be a valuable predictor of GHD. Significantly low age-adjusted levels of IGF-I are notably indicative of GHD, but the presence of normal or elevated IGF-I levels does not preclude the diagnosis (51–56).

The diagnostic accuracy of measuring IGF-I concentrations as an alternative to provocative testing to recognize GHD has been compared with receiver operating characteristic (ROC) analysis. Corneli et al. reported that the best pair of highest sensitivity (96.6%) and specificity (74.6%) for identifying GHD was obtained with an IGF-I cutoff of −1.3 SDS (57), whereas Maghnie et al. found the best combination of sensitivity (77%) and specificity (100%) with a cutoff of −1.7 SDS (58). In the current study, an IGF-I concentration cutoff of −1.4 SDS relative to the age-adjusted means of the reference sample was used to determine a positive screen for GHD. The high specificity of IGF-I measurements reduces the likelihood of false positives, but the low sensitivity of the measurements suggests that some participants with GHD may not have been identified.

Insufficient GH secretion in adults has been shown to have negative effects in several cognitive domains and to be associated with numerous behavioral symptoms (17, 59, 60). These include reduced physical mobility, fatigue, sleep difficulties, depression, social isolation, low sexual drive, lowered metabolic rate, and reduced aerobic capacity (61, 62). Poor quality of life is also strongly associated with adult GHD, especially in terms of vitality and energy (63–66). Adult GHD results in reduced lean body mass, lipidemia, and increased adiposity. Even partial GHD is associated with adverse lipid profiles and early atherosclerosis in adult patients (67).

The mTBI group included two veteran participants who were considered hypogonadal based on our criteria: a total testosterone concentration less than the fifth percentile of the reference sample together with an LH or FSH level below the 10th percentile reference level. Hypogonadism has deleterious effects beyond those on fertility, psychosexual function, and general well-being. Male testosterone deficits are associated with muscle weakness, reduced lean body mass, decreased energy and motivation, impaired exercise tolerance, and premature mortality secondary to cardiovascular disease (68, 69).

One mTBI participant, mTBI-E, exhibited a highly elevated concentration of serum prolactin, 2.5 times higher than the next highest concentration measured in either group. Hyperprolactinemia is causally associated with hypogonadism through attenuation of LH and FSH secretion and desensitization of gonadal LH and FSH receptors. Reduction of FSH levels by excessive prolactin secretion also gives rise to hypoactive sexual desire and erectile dysfunction. The symptoms of hyperprolactinemia include gynecomastia and erectile dysfunction in men, irregular menses in women, and decreased libido, infertility, galactorrhea, and osteoporosis in both sexes (70, 71).

Prolactin is the only anterior pituitary hormone for which secretion is under primarily inhibitory control. Dopamine tonically suppresses prolactin secretion, and diminishment of this inhibition results in high levels of circulating prolactin. Antipsychotic medications that act as antagonists at dopamine D2 receptors may induce hyperprolactinemia (70). Participant mTBI-E had been taking quetiapine, a so-called dopamine-sparing antipsychotic (72). Though less likely to elevate prolactin levels than other antipsychotics (e.g., haloperidol and risperidone) hyperprolactinemia prevalence rates of up to 29% have been reported in association with its use (73, 74).

Participant mTBI-E had also been taking prazosin, an α-1 adrenoceptor antagonist. Norepinephrine, as well as dopamine, has been shown to inhibit prolactin secretion in sheep *via* pituitary α-1 receptors both *in vivo* and *in vitro* (75). Use of quetiapine and/or prazosin may have been responsible for the elevation of prolactin levels in this individual. Participant DC-C, who was found to have a dopamine level fractionally above the 95th percentile of the reference group, reported taking only natural supplements.

Two veterans in the mTBI group screened positive for central hypothyroidism, and no members of either group were found with sAI. TSH and ACTH deficiencies have been less frequently reported in previous studies of pituitary dysfunction after TBI than those of gonadotropins or GH (66, 76, 77). This outcome may be due in part to the anatomical location of thyrotrophs and corticotrophs in the pituitary’s protected median wedge. Vascular input to this region is carried by both long hypophyseal portal vessels and by the inferior hypophyseal artery *via* the short hypophyseal portal vessels. In contrast, GH-secreting somatotrophs are more susceptible to damage because they are situated in the gland’s exposed lateral wings and are almost exclusively dependent on the long hypophyseal portal vessels for their blood supply. Gonadotrophs are distributed throughout the anterior pituitary (78).

A large majority of studies of chronic PTHP have focused solely on anterior pituitary hormonal disorders. Of those studies that have included investigations of the deleterious effects of TBI on posterior pituitary function, most have reported the existence of disorders related to that lobe as well, the most common being DI (15, 22, 66, 77, 79–81).

In the current study, in addition to the 12 participants from both groups who screened positive for anomalous anterior pituitary hormone levels, five veterans with mTBI (including one growth hormone deficient and one hypothyroid individual) screened positive for posterior pituitary hormone deficiencies. Repeated assays for vasopressin gave inconsistent results and the data were deemed to be unreliable. A positive screen for DI was defined by a combination of USG less than or equal to 1.005, urine osmolality less than 200 mOsm/kg, and normal Posm.

Oxytocin levels below the fifth percentile threshold of the reference group were found in plasma of mTBI-H and mTBI-L. Apart from its important role in parturition and lactation, there is no strong evidence for the clinical significance of oxytocin deficiency. However, many animal and human studies have provided evidence for the positive association of oxytocin levels with behavior and attitudes related to social interactions such as maternal and romantic bonding, attenuation of stress responses, and mediation of social support (82, 83). Deficiencies in oxytocin appear to be associated with psychiatric conditions involving deficits in social behavior including autism spectrum disorders and schizophrenia (84–86). Although findings in the literature lack consistency, recent research on both central and peripheral actions of oxytocin have identified potential relationships with mood and anxiety (87), learning and memory (88), improvement of wound healing (89), modulation of inflammatory responses (90, 91), neuroprotection (89, 91, 92), regulation of food intake and body weight (93), and reduction of pain sensitivity (94, 95).

There was no significant difference between the numbers of blast exposures sustained by the two mTBI subgroups during Iraq/Afghanistan deployment (Table 4). Two-way ANOVA revealed significant main effects of both mTBI and hypopituitarism on the scores of the four groups on self-report questionnaires measuring PTSD (PCL-M), and postconcussive symptoms (NSI). This finding suggests an independent effect of hypopituitarism on behavioral measures.

In another study currently in progress, the blast-related concussion histories of the two mTBI groups are similar to one another, with the mTBI-N group tending to be higher than the mTBI-HP group in number of deployment mTBIs (96). Nonetheless, the TBI-HP group endorses significantly greater symptom frequency and severity than the TBI-N group on the PCL-M, NSI, PHQ-9, and the PSQI described above, as well as the Quality of Life Assessment of GHD in Adults (QoL-AGHDA) (97), and the Fatigue Severity Scale (96, 98). These preliminary findings support the hypothesis that mTBI-induced hypopituitarism has an additive effect in increasing neurobehavioral symptomatology beyond that of mTBI alone. There is an expanding literature about the wide-ranging and severe consequences of repetitive concussions (22–26), but there is thus far no evidence from our studies that the number of concussions is related to the prevalence of hypopituitarism.

There have been very few published studies of PTHP after blast-related concussion to date (21, 27, 99, 100) other than our preliminary report. Baxter and colleagues found that six of 19 (32.0%) Afghanistan-deployed male United Kingdom soldiers with moderate or severe blast-related TBI had anterior pituitary dysfunction, compared to one of 39 (2.6%) age- and gender-matched civilian control subjects with moderate-to-severe non-blast TBI (99). In another study, 5 of 20 veterans with mTBI sustained during combat (85% from explosive blast) had sub-threshold GH responses to glucagon administration (100). A study using an integrated structural magnetic resonance imaging protocol examined 834 military service members with TBI. The participants were diagnosed with primarily chronic (mean, 1,381, median, 888, days after injury), blast-related (84%), mild (92%) TBI. The results showed that 29.0% of military TBI participants had pituitary abnormalities compared with only 2.4% in a group of 42 control participants. The rates included all pituitary abnormalities noted in structural images, both before and after administration of contrast agent (21).

There is significant overlap in the symptomology of persistent postconcussive (PPCS) and PTHP, and similarity of both to PTSD. This congruity of symptoms increases the difficulty of deciphering the etiology, progression, and identifiable differences among the conditions that are essential for successful treatment, recovery, and rehabilitation (90). Lack of recognition of pituitary dysfunction may be conducive to diminished quality of life, fatigue, cognitive deficits, sleep difficulties, sexual dysfunction, psychiatric and behavioral disorders such as anxiety, irritability, depression, and social isolation, as well as deleterious modifications in body composition and metabolism leading to increased cardiovascular mortality. Unlike PTSD and PPCS, PTHP is often readily treatable once identified, and its symptoms may often be reversed or attenuated with appropriate hormone replacement therapy.

Routine endocrine evaluation of hypopituitarism after brain injury has been advocated by several investigators studying PTHP (25, 53, 76, 77, 80, 101–108). A recent review of the literature stated that because “many of the symptoms of hypopituitarism are similar to those of TBI, it is important to make clinicians caring for combat veterans aware of its occurrence … All patients who had a TBI of any severity, should undergo baseline hormonal evaluation” (109). The results of this study further support the need for endocrine evaluation in military personnel and veterans with a history of blast-related mTBI who are currently reporting postconcussive symptoms.

## Ethics Statement

The Congressionally Directed Medical Research Programs Concept Award that funded this study prohibited direct sampling of biological fluids from human participants and access to identifiable private information. Therefore, all plasma and serum samples, demographic, and blast exposure data used in the study were obtained from an established biorepository entitled “Alzheimer’s Disease Research Center Participant Registry and Sample Repository.” The VA Puget Sound Health Care System Institutional Review Board and the US Army Medical Research and Materiel Command Office of Research Protections Human Research Protection Office approved the subject protocol with a waiver of informed consent. All participants whose samples were utilized had consented to have their samples and data used in future research of this type. No direct intervention with any participant was allowed. These conditions precluded the use of provocative testing as a part of the screening procedure.

## Author Contributions

CW, EP, and KP contributed to the conception and design of the study. AU wrote the first draft of the manuscript. CW and JS wrote the remaining drafts and final manuscript. JS and AU compiled and organized the data. JS performed statistical analyses and literature searches and prepared the tables and figures. EC and CS performed the hormone assays and compiled the hormone data. All authors contributed to manuscript revision and read and approved the submitted version.

## Conflict of Interest Statement

The authors declare that the research was conducted in the absence of any commercial or financial relationships that could be construed as a potential conflict of interest.

## Abbreviations

ACTH, adrenocorticotropin; AUDIT-C, alcohol use disorders identification test-consumption; ACRM, American Congress of Rehabilitation Medicine; ANOVA, analysis of variance; BUN, blood urea nitrogen; BMI, body mass index; CAPS, clinician-administered PTSD scale for DSM-IV; CEQ, combat experiences questionnaire; DC, deployment control; DI, diabetes insipidus; DSM-IV, diagnostic and statistical manual of mental disorders, fourth edition; EIA, enzyme immunoassay; ELISA, enzyme-linked immunosorbent assay; EDTA, ethylenediaminetetraacetic acid; FSH, follicle-stimulating hormone; GHD, growth hormone deficiency; IRMA, immunoradiometric assay; IGF-I, insulin-like growth factor-I; LOC, loss of consciousness; LH, luteinizing hormone; mTBI, mild traumatic brain injuries; NSI, neurobehavioral symptom inventory; PHQ-9, patient health questionnaire-9; PCTL, percentile; PPCS, persistent postconcussive; PSQI, Pittsburgh Sleep Quality Index; Posm, plasma osmolality; PTHP, posttraumatic hypopituitarism; PTSD, posttraumatic stress disorder; PCL-M, PTSD checklist-military version; ROC, receiver operating characteristic; RIA, radioimmunoassay; sAI, secondary adrenal insufficiency; SDS, SD score; SIADH, syndrome of inappropriate antidiuretic hormone secretion; TSH, thyroid-stimulating hormone; TMT, trail making test; Uosm, urine osmolality; USG, urine specific gravity; WTAR, Wechsler test of adult reading.
